# Fishers’ Behaviour in Response to the Implementation of a Marine Protected Area

**DOI:** 10.1371/journal.pone.0065057

**Published:** 2013-06-03

**Authors:** Bárbara Horta e Costa, Marisa I. Batista, Leonel Gonçalves, Karim Erzini, Jennifer E. Caselle, Henrique N. Cabral, Emanuel J. Gonçalves

**Affiliations:** 1 Eco-Ethology Research Unit, ISPA – Instituto Universitário, Lisboa, Portugal; 2 Centre of Marine Sciences - CCMAR, University of Algarve, Campus de Gambelas, Faro, Portugal; 3 Centro de Oceanografia, Faculdade de Ciências, Universidade de Lisboa, Lisboa, Portugal; 4 Marine Science Institute, University of California, Santa Barbara, California, United States of America; Leibniz Center for Tropical Marine Ecology, Germany

## Abstract

Marine Protected Areas (MPAs) have been widely proposed as a fisheries management tool in addition to their conservation purposes. Despite this, few studies have satisfactorily assessed the dynamics of fishers’ adaptations to the loss of fishing grounds. Here we used data from before, during and after the implementation of the management plan of a temperate Atlantic multiple-use MPA to examine the factors affecting the spatial and temporal distribution of different gears used by the artisanal fishing fleet. The position of vessels and gear types were obtained by visual surveys and related to spatial features of the marine park. A hotspot analysis was conducted to identify heavily utilized patches for each fishing gear and time period. The contribution of individual vessels to each significant cluster was assessed to better understand fishers’ choices. Different fisheries responded differently to the implementation of protection measures, with preferred habitats of target species driving much of the fishers’ choices. Within each fishery, individual fishers showed distinct strategies with some operating in a broader area whereas others kept preferred territories. Our findings are based on reliable methods that can easily be applied in coastal multipurpose MPAs to monitor and assess fisheries and fishers responses to different management rules and protection levels. This paper is the first in-depth empirical study where fishers’ choices from artisanal fisheries were analysed before, during and after the implementation of a MPA, thereby allowing a clearer understanding of the dynamics of local fisheries and providing significant lessons for marine conservation and management of coastal systems.

## Introduction

Besides conservation purposes, marine protected areas (MPAs) have also been suggested as important fisheries management tools [1]–[3]. The expected effects from the exclusion of extractive activities in marine reserves (no-take) are an increase in abundance, size and fecundity of fished individuals, especially for those most impacted by fisheries [4]. This so-called “reserve effect” is expected to translate to biomass export of post-settlers to adjacent areas (spillover) which may, in turn, depend on density-dependent mechanisms and carrying capacity of protected and adjacent areas, as well as connectivity of suitable habitats [4], [5]. Some authors have also suggested that fisheries are more likely to benefit through larval export from reserves to surrounding areas due to an increase in size and fecundity of adults inside the reserve [4], [6], but these benefits have been much more difficult to detect [7], [8]. Further to these direct responses, indirect effects may also occur and affect nearby areas after some time due to the build-up of top-predators and subsequent trophic cascades inside no-take areas [9], [10].

While some of these effects are well documented, their magnitude depends not only on factors such as habitat connectivity, oceanographic characteristics, species life histories, environmental requirements and mobility patterns [10], [11], but also on the enforcement of rules and compliance by local users [12]. Several reviews have focussed on the evaluation of the reserve effect [2], [9], [13], but fewer studies have empirically considered the patchy distribution of species and fishing effort [8], [14]–[16], which might have a large influence on the assessment of fisheries benefits of a MPA. In fact, the loss of fishing grounds and the redistribution of fishing effort in adjacent areas may affect the magnitude of the reserve effect [12]. Hence, it is important to include and understand fishers’ behaviour in relation to enforced management rules, habitat preferences of commercial species and other fishers or competing activities.

The concentration of fishing effort near boundaries of no-take areas (i.e. fishing-the-line) is not uncommon and can be interpreted as spillover benefits to adjacent fisheries [16], [17]. On the other hand, very intense fishing-the-line behaviour may produce a sharp decrease in density adjacent to a reserve boundary [8]. This is intrinsically related to gear selectivity since species catchability influences the extent of spillover and the effects inside the reserve [16]. Traditional fishing grounds and travel costs may also influence fisheries allocation [18]. Recently, some studies have shown that the distance to borders of no-take areas, water depth and distance to the landing port are the most important factors explaining fisheries aggregations around MPAs, which can be associated, respectively, with fishery benefits, target species distribution and costs [14]–[16], [19], [20]. The responses of coastal [21], [22] and recreational [23] fisheries distribution before and some years after rezoning have been reported for tropical MPAs. Fisheries displacement was assessed mainly based on face-to-face interviews, and the direct observations conducted after rezoning in one of the studies showed that fishers were reluctant to self-report spatial infringements [21]. Therefore, in spite of work on the redistribution of fishing effort in large-scale trawl fisheries [14], [24], there are no empirical studies using direct observations to compare spatial fishing allocations before and after implementation of protection measures in coastal MPAs where artisanal fisheries dominate.

Here we provide the first in-depth assessment of spatial redistribution of fishers in response to MPA implementation. The Arrábida Marine Park is a multiple-use MPA containing a core no-take zone surrounded by several zones with intermediate levels of protection where some human activities are allowed (e.g. small-scale fisheries, diving, tourism and recreational fisheries). In this coastal area artisanal fisheries prevail, where fishers use multiple gears, including trammel and gill nets, traps, longlines and jigs [25]. This study aims to analyse density patterns of the main fishing gear types by comparing the spatial distribution of vessels and buoys before, during and after implementation of the MPA management plan. Density clusters of individual fishers in preferred fishing grounds were investigated through time to understand fishers’ choices and adaptability to the MPA rules.

## Methods

### Study Area

**Figure 1:**
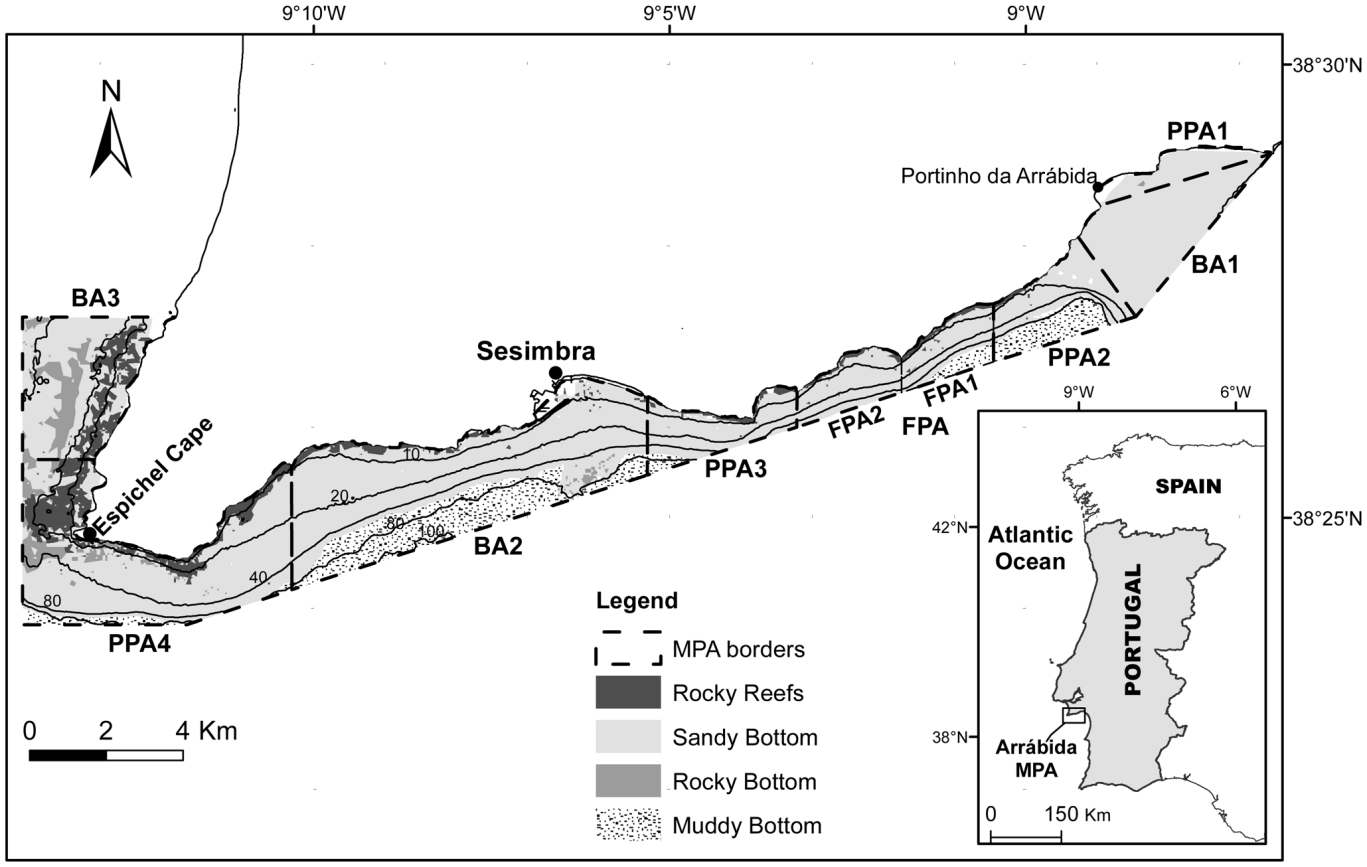
Map of the Arrábida Marine Park with zoning implemented by the management plan. Zoning: BA – Buffer areas; PPA – Partially-protected areas, FPA – Fully-protected area (divided in FPA1 and FPA2 due to the transitory phase of the management plan implementation – see Methods). Bathymetry and main habitat types are shown [26].

The Arrábida Marine Park (AMP) is a 38 km stretch of coastline (53 km^2^) on the west coast of Portugal, adjacent to a terrestrial nature park created in 1976– the Arrábida Nature Park. The marine park includes the rocky shores and adjacent mixed sandy substrata between north of the Espichel Cape (38°27′N, 9°12′W) and Portinho da Arrábida (38°29′N, 8°57′W) (Figure 1). This area is utilized year-round for commercial and recreational activities as it faces south and is protected from the prevailing north and northwest winds and waves. Nearby are the cities of Lisboa and Setúbal, the latter being an important fishing and commercial port located to the east of the park in the Sado estuary. In the middle of the park there is a small fishing town, Sesimbra, which has a long fishing tradition and is nowadays an important touristic area.

Nearshore, the subtidal shallow rocky reefs are dominated by boulders created by the erosion of the calcareous cliffs and by bedrock with fissures and crevices generating a complex habitat. This habitat is confined to the first 100–150 m from shore except on the west tip of the park where reefs extend beyond this range. Sand is the primary habitat covering the majority of the park from shallow (adjacent to rocky reefs and rocky outcrops) to deeper areas where it is replaced by mud.

The management plan was approved in August 2005 and multiple areas with differing levels of protection have been designated (Figure 1): a fully-protected area (FPA) totalling 4 km^2^; four partially-protected areas (PPA) totalling 21 km^2^; and three buffer areas (BA) encompassing 28 km^2^. Commercial diving for bivalves or other marine organisms, spearfishing, trawling, dredging and purse seining are forbidden in the whole park. These activities were considered to be the ones with the largest impact on coastal marine communities. Commercial fishing licenses for the park were exclusively allocated to fishers from Sesimbra who owned vessels smaller than 7 m in length. The FPA is a no-take, no-go area (except for research, monitoring and education purposes). In the PPAs, artisanal fishing with traps and jigs is allowed, but only beyond 200 m from coast and no extractive recreational activities (i.e. angling) are permitted. In the BAs, licensed fishing vessels and authorised recreational fishing are allowed.

The park’s management plan established a transitional period for fisheries, aimed at facilitating the adaptation by fishers to the changes in uses, in which the rules of the different protection zones were gradually implemented during the first four years. In August 2006, management measures were enforced in the BAs, the east half of the FPA (FPA1) began as a PPA and the Portinho PPA (PPA1) was implemented. One year later, the remaining PPAs were implemented and the west half of the FPA (FPA2) started as a PPA. In 2008, the east half of the (current) FPA changed from PPA to FPA. The west half of the FPA was enforced in the summer of 2009, ending the transition period (Portuguese legislation, Council of Ministers Resolution 141/2005) (see Figure 1).

The zoning and rules of the marine park were submitted to a consultation process as required by the Portuguese Law. This process involved NGOs, local authorities, professional fishers associations and other stakeholders. However, there is generally a low level of representation of artisanal fishers using small vessels in the fishers associations. This created problems in understanding the park objectives and accepting the management rules and it is still a focus of mistrust not only between the fishers association and the park authority, but also among fishers themselves. The exclusion of larger vessels from the Park was also very contentious, since the associations represent mainly these fishers. Zones were decided based on the MPA objectives and natural values present, with fishers’ perceptions not influencing the zoning scheme. However, the initial plan (before consultation) was greatly changed to address the artisanal fishers’ concerns, namely by including nets in the BAs (no nets were to be allowed in the MPA in the initial proposal) and reducing the level of protection in the PPAs with traps and jigs being allowed beyond 200 m from coast (in the initial proposal no fishing activities were considered in the PPAs). Nowadays, fishers with license to operate within the marine park appear to generally support it [26], possibly due to the decrease in fishing effort from competing gears (e.g. dredges) and larger vessels, but also to the exclusion of other competing fishing activities, such as spearfishing. However, most seem to disagree with several measures and enforcement strategies (depending on which type of gear they use), although poaching inside the no-take area is not supported, which suggests recognition of the benefits this area may provide.

### Sampling Surveys

Fishing vessels and buoys within the marine park limits were surveyed along transects by boat. During each sampling day (one sample), the location of vessels and buoys (using a Global Positioning System - GPS), fishing gear type and vessels’ names were recorded for all vessels and fishing buoys surveyed (the Portuguese legislation requires that fishing buoys at sea have to be identified with a code for fishing gear type and vessel identification). Two transects were performed each day covering the entire marine park (except the area north of the Espichel cape due to frequent rough sea conditions). The first transect, focussed on vessels, started early (6∶45 to 7∶45 am) in the east part of the Park, near the Portinho da Arrábida bay, and ended at Espichel cape. All buoys were then surveyed on the second transect in the opposite direction. Sampling was carried out inside the Arrábida Marine Park under a permit by the marine park authority (Parque Natural da Arrábida, Instituto da Conservação da Natureza e da Biodiversidade).

Sampling was carried out in five different periods corresponding to the ‘before’, ‘implementation’ and ‘after’ phases of the management plan (see above): ‘Before’ period – from April to November 2004 (7 samples for buoys and 28 samples for jig vessels); ‘Implementation’ period refers to Years 1, 2 and 3: Year 1– from March to August 2007 (15 complete samples: for both buoys and vessels), Year 2– September 2007 to February 2008 (14 complete samples), Year 3– November 2008 to August 2009 (16 complete samples); ‘After’ period – September 2009 to December 2009 (6 complete samples). This classification was used for all analyses. Buoy surveys in the Before period were not uniformly distributed over time, whereas in the Implementation and After periods an average of three and two samples/month were conducted, respectively. The small vessels using jigs were only identified in Year 3 and in the After period.

In the Before period, vessel surveys were shore based, with ten stations established on the high cliffs along the coast covering the entire marine park. Sampling was done early in the morning on a weekly basis, and vessels were georeferenced based on the topographic triangulation method [27], using an electronic theodolite (Topcon, model DT –30) and a GPS. This method has a high level of accuracy in terms of the spatial positioning of objects/features [28].

Three fishing gear types were analysed since they were identified as the most important in the study area: traps, trammel and gill nets, and jigs. Other fishing gears were recorded but were observed infrequently (longlines) or occurred only before the management plan was approved (purse seines). Data for vessel location was used for jigs, since this gear is operated manually directly below the fishing vessel, while buoy geographic coordinates were used for stationary gear (traps and nets).

Even though some vessels were seen few times or only in one of the periods (some fish infrequently, others did not maintained their license or were transferred to other ports), others were observed consistently over the course of the study, with some of them fishing with more than one gear. Three vessels were detected fishing with the three gear types, one with jigs and traps and twenty with nets and traps. Fifty four vessels were seen fishing exclusively with traps, fourteen with nets and a hundred and thirteen vessels were fishing only with jigs.

### Data Analysis

#### Generalized additive models

For all analyses data were aggregated by periods and the three fishing gear types. The spatial and temporal fishing dynamics in the AMP and possible explanatory variables were analysed combining geographic information system (GIS) techniques and generalized additive models (GAMs). The marine park limits and zoning (source: AMP authority) were superimposed onto a map of habitats and bathymetry (source: [26]) using a 500×500 m grid (0.25 km^2^ cells), although some grids were smaller due to the coastal line and legal borders. Densities (counts per area) of the main fishing gears in the park were summarized by grid and for GAMs only fished grids (with recorded fishing activities) were included [15], [16].

Fishing effort allocation was related to the following features using GIS to measure the shortest linear distance (m) from each feature to the mid-point of each grid: distance to Sesimbra port (DistPort), distance to coast (DistCoast), depth, distance to the partially-protected areas (DistPPA), distance to the fully protected area (DistFPA), distance to the 200 m line offshore of the coast (Dist200 m) and distance to the ¼ nautical mile line (Dist1/4 nm). The variable Dist200 m was only related to traps and jigs since it is a limit implemented inside the PPAs, where nets are excluded. Therefore, when these gears were used beyond the 200 m limit but inside the PPAs, the distance to the borders of these areas was negative, to distinguish from fishing gears operating outside these areas. On the other hand, Dist1/4 nm is a national legal limit only for bottom fixed nets (trammel nets and gillnets). The DistPPA1 (PPA1 refers to Portinho bay) was removed from the analyses since only forbidden small drift nets were found fishing there before the management plan started. Throughout the implementation period (Years 1, 2 and 3), DistPPA and DistFPA refer to the respective regime of each protected area in each period.

GAMs were used to explore the density response to the explanatory variables as non-linear relationships were expected and this non-parametric technique does not require linear trends [29]. Autocorrelations among spatial features were tested for each period and only variables with no or low levels of correlation were used to conduct these models. Choosing gamma as the exponential family and using a square root transformation of the response variable resulted in residuals showing a good approximation with normality. Several GAMs were run to test for the best fitted model for each gear type and period. Since some variables were highly correlated we selected those considered to better explain fishers’ choices: DistPort, depth, DistPPA and DistFPA. Additionally, alternative models were run to test the influence of the current FPA (DistFPA) during the Before, Year 1 and Year 2 periods (i.e. before full protection was implemented) to evaluate if this was an area pre-selected for its specific characteristics. All explanatory variables included in the models were allowed to be non-linear (using smoothers). Approximate significance of the smooth terms and deviance explained were obtained from each GAM.

For all gears, depth was highly correlated to distance to the coast. For traps and jigs, depth was also highly correlated to Dist200 m and for nets to Dist1/4 nm. Therefore, significant results for depth should be interpreted with caution as they may also reflect significant effects of those other variables. Additionally, a bottom type was assigned to each grid cell using habitat maps. Bottom type by grid cell was classified into the following categories: sand, mud, rock (isolated rocky outcrops) and reefs (coastal shallow rocky reefs). Variables related to habitat were not included in GAMs due to co-linearity but since bottom type may influence both species and fishers’ distribution, a Kruskal–Wallis test was conducted to assess the density of gear types (square root transformed) relative to bottom type in each period. Multiple comparison tests evaluated differences in density for each pair of habitat-types. These analyses were conducted with the R 2.14.1 software [30].

#### Spatial hotspot analyses

Fishing areas were analysed using area pattern statistics [31]. Specifically, hotspot analysis was performed in order to study the changes in uses in the main locations chosen by fishers across the five periods, for each fishing gear type. Spatial patterns were investigated using GIS modelling techniques with Arcgis 10.0 (ESRI) software. For this, a 250×250 m grid covering the marine park was superimposed to the fishing GPS points. Hotspot analysis was conducted separately for each of the main gear types with the geographic positions of each vessel occurring in each grid with the aim to study the patterns of use of fishing grounds by individual fishers. To determine statistically significant hotspots, Getis-Ord Gi* statistic (which gives a Z-score and a p-value) [32] was calculated for each grid cell. Statistical tests for significant spatial patterns in data (obtained by a Z score, which varied between -1.96 and +1.96), were compared with the null hypothesis of complete spatial randomness (CSR) with a 95% confidence level against the alternative hypothesis that events are spatially clustered or dispersed. The larger the Z score, the more intense is the clustering of high values (i.e. a hotspot) whereas for negative Z scores, the smaller the Z score, the more intense is the clustering of low values (cold spot) [33]. Significant clusters were defined as the aggregation of adjacent grid cells with a Z-score ≥ |1.96|, consistent with spatial clustering. To understand the vessel composition in each aggregation, the number of vessels and their percent contribution to each significant cluster was calculated by period and fishing gear. However, since the identification of individual vessels using jigs was not always possible in the Before, Year 1 and Year 2periods, the contribution of these vessels was only evaluated for clusters from Year 3 and the After period.

To perform these analyses, the best distance band was chosen based on global Morans I statistics for spatial autocorrelation [33]. This tool provides a Z-score for the entire study area, measuring spatial autocorrelation based on feature locations and attribute values. To calculate Morans I, the 200 meters distance was used as the starting distance with a cut-off at 800 meters. The minimum distance was chosen based on grid size and the maximum observed dispersion of points. The conceptualization of spatial relationships used for the analysis was the zone of indifference. The final global Z-scores were plotted against the Euclidean distance values and when the increase of the distance caused a decrease in the Z-value (peak), that distance was selected as the best distance band to use in the hotspot analysis [33].

## Results

### Traps

The selected models for the density of traps by period explained between 16.5% and 53.2% of the total variability (Table S1). Overall, the distance to port significantly influenced fisher’s behaviour in the Before period and Year 1 (p<0.05), whereas depth influenced effort density allocation in all periods (Year 1: p<0.05; Years 2, 3, After: p<0.001), although in the Before period it was marginally non-significant (p = 0.055). The distance to PPAs was not significant in all periods but, interestingly, after the two halves of the fully protection zone were implemented (in Year 3 and After periods), the distance to their borders significantly influenced the variance (Year 3: p<0.01; After: p<0.001).

In separate models (not shown), distance to the current FPA was tested for the periods before this protection level was effective (Before, Year 1 and Year 2). This variable did not influence the density of traps in the Before period but significant differences were found in both Year 1 (p<0.05) and Year 2 (p<0.001).

**Figure 2:**
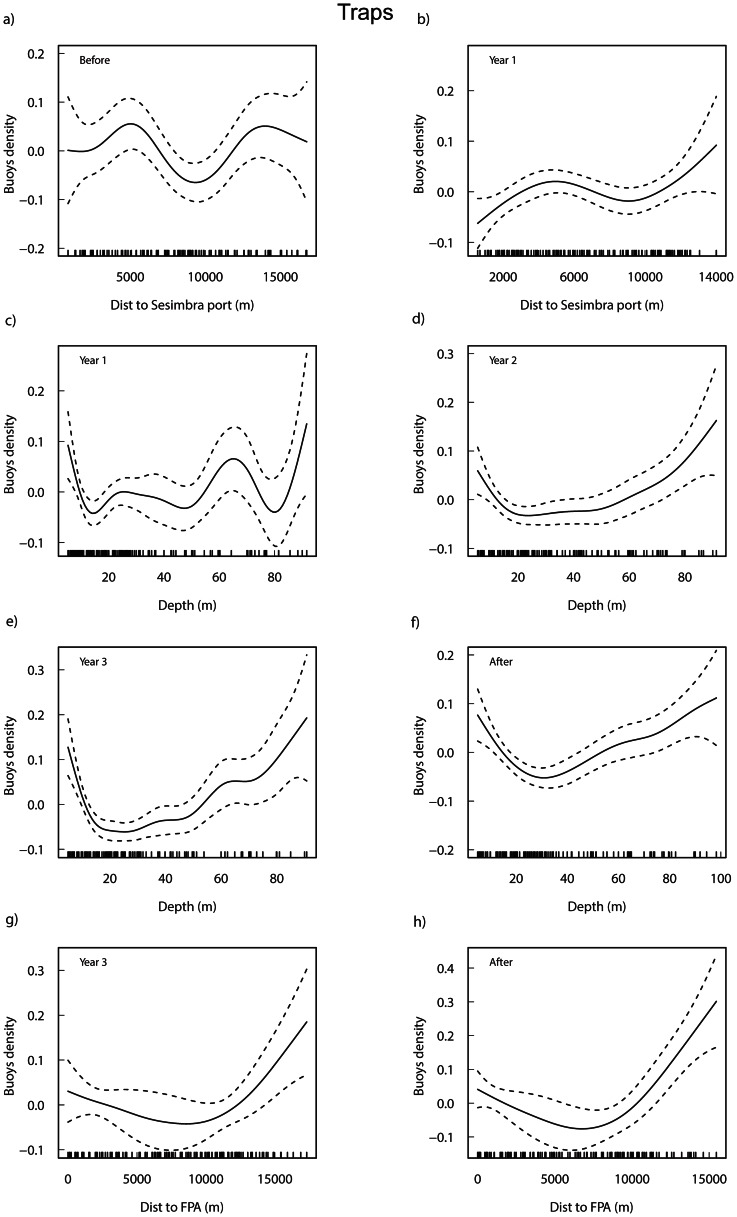
Additive fits of the significant predictor variables to the density of trap buoys for each period. Distance to Sesimbra port (a, b), depth (c–f), and distance to FPA (g, h) from the significant periods of the selected GAMs (see Table S1) are shown. Tick marks above the *x*-axis indicate the distribution of observations and the *y*-axis shows the contribution of the smoother to the fitted values. The solid line is the estimated smoothing curve and the dotted lines show the 95% confidence intervals.

The additive fits of the significant predictor variables from all modelled time periods are shown in Figure 2. During the Before and Year 1 periods the density increased with distance to port showing two peaks, at around 5000 m and 13000 m (Figure 2a, b). Trap density decreased steeply with depth up to approximately 18–20 m, and then increased up to approximately 80–90 m, although there are few observations at those deeper locations (Figure 2c–f) which were mainly situated in front of Sesimbra port where depth increases rapidly offshore (Figure 1). The density of traps also decreased with distance to the fully protected area (Figure 2g, h), but this trend shifted at around 8000 m from the FPA border, where density started to increase.

Density patterns were not influenced by habitat type in each of the five periods. However, aggregating data from all periods showed a significant effect of habitat on effort density allocation (Kruskal-Wallis chi-square = 16.6, p<0.001). Multiple comparisons revealed significantly (p<0.05) higher density of traps in sand compared to mud and rock, but not compared to reefs.

### Nets

The selected models for the density of nets by period explained between 37.6% and 64.30% of the total variability (Table S2). Overall, distance to port (except in Year 3; Before, Year 2: p<0.001; Year 1: p<0.005; After: p<0.01) and depth (p<0.05; except in Year 3 and the After period) had an important role in the spatial allocation of nets. Additionally, distance to PPA and to FPA started to have a significant influence in Year 2 and in the After period, respectively (p<0.05). Unlike traps, in the models testing the distance to the current FPA (not shown) for the periods before this protection level was effective, this descriptor was significant for the density of nets before the management plan was implemented (p<0.01) but lost significance after its implementation.

**Figure 3:**
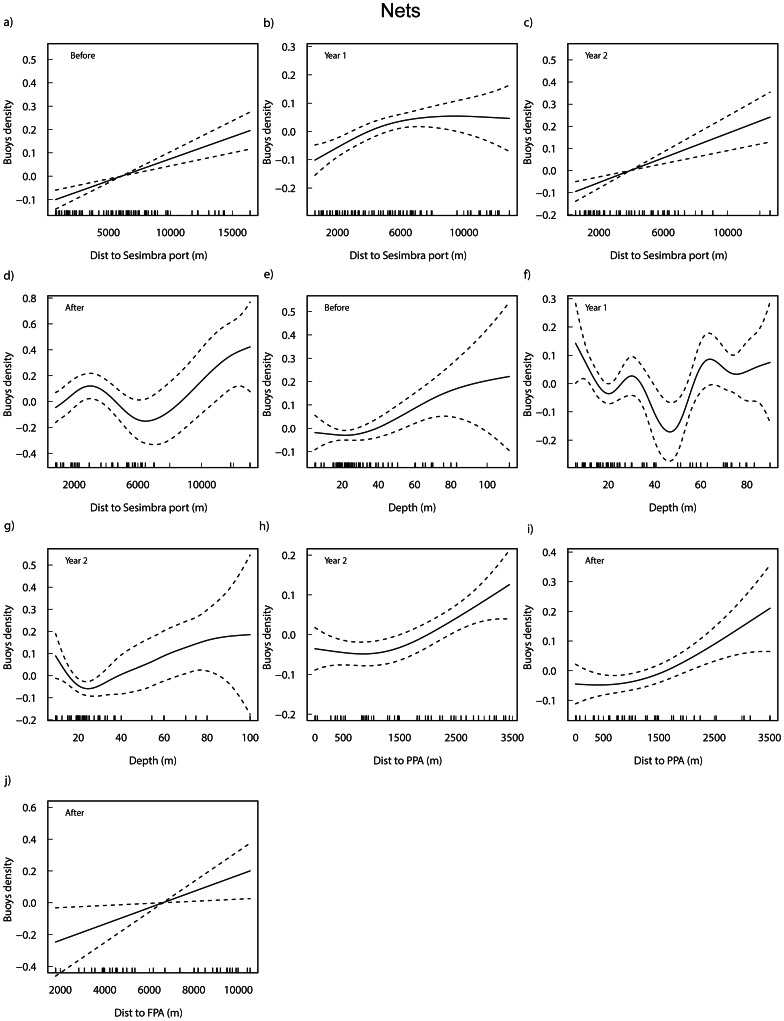
Additive fits of the significant predictor variables to the density of nets buoys for each period. Distance to Sesimbra port (a–d), depth (e–g) and distance to PPA (h, i) and to FPA (j) from the significant periods of the selected GAMs (see Table S2) are shown. Tick marks above the *x*-axis indicate the distribution of observations and the *y*-axis shows the contribution of the smoother to the fitted values. The solid line is the estimated smoothing curve and the dotted lines show the 95% confidence intervals.

The density of nets increased with the distance to port (Figure 3a–d) although there are few observations beyond 10000 m (Before), 8000 m (Year 1) and 6000 m (Year 2). In the After period, there was a decrease in density between 3000 m and 6000 m. Nets generally decreased with depth up to approximately 20 meters, increasing afterwards (Figure 3e–g). In Year 1 a steep decrease in density was found at around 50 m. Density in relation to distance to PPA increased significantly in Year 2 (and was marginally non-significantly in Year 3) and in the After period, when distance to FPA also increased significantly (Figure 3h–j).

Habitat type was significantly related to the density of nets only in Year 3 (Kruskal-Wallis chi-square = 9.4, p<0.05), with multiple comparisons showing that density on rock was higher than on mud (p<0.05), with reefs and sand showing intermediate values. When aggregating data from all periods (Kruskal-Wallis chi-square = 12.9, p<0.005) the same pair of habitats differed significantly (p<0.05).

### Jigs

The selected models for the density of vessels fishing with jigs show a very high deviance varying between 56.6% and 84.4% which was much higher than that of the other gear types (Table S3). Depth was a highly significant factor associated to the density of jigs in all periods (p<0.001; except in Year 1 when it was marginally non-significant). In both Year 3 and the After period, it was the only significant factor in the model. Distance to port was also an important factor both in the Before (p<0.05) and Year 2 (p<0.001) periods. The only period where protection measures significantly influenced the density of jigs was in Year 1 (p<0.05) with a decreasing pattern with the distance to PPA. No significant influence was found on jigs allocation in relation to the distance to FPA. When the distance to the current FPA was tested for the first periods (Before, Year 1 and Year 2) in separated models (not shown) it was also highly significant before protection started (p<0.001) and in Year 2 (p<0.005).

**Figure 4:**
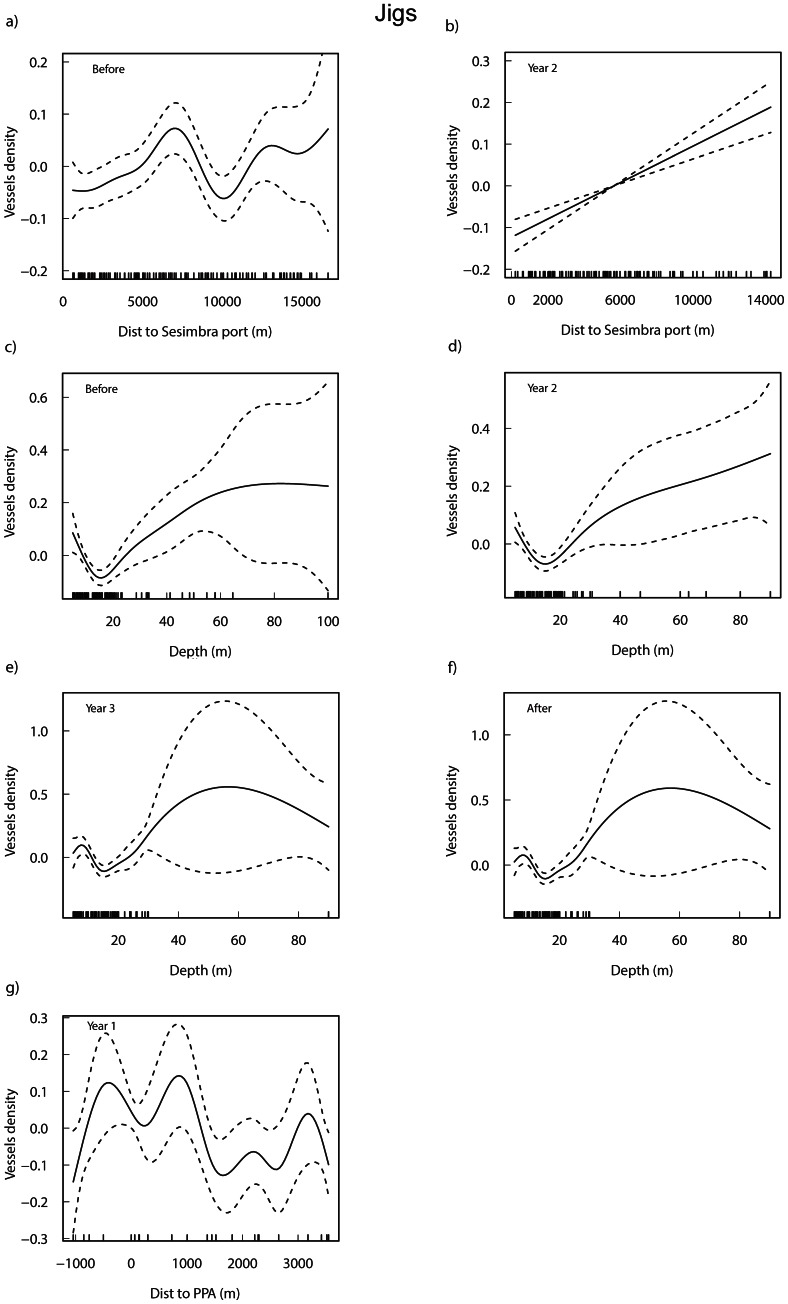
Additive fits of the significant predictor variables to the density of jig vessels for each period. Distance to Sesimbra port (a, b), depth (c–f), and distance to PPA (g) from the significant periods of the selected GAMs (see Table S3) are shown. Tick marks above the *x*-axis indicate the distribution of observations and the *y*-axis shows the contribution of the smoother to the fitted values. The solid line is the estimated smoothing curve and the dotted lines show the 95% confidence intervals. Negative distances refer to jig vessels fishing inside the PPA.

Overall, jig density increased with the distance to port, especially in the first 5000–6000 m (Figure 4a, b). Additionally, in the Before period there was a decrease in density between 7000 m and 10000 m followed by a second increase further away from port. In the following periods, very few vessels were seen beyond 8000 m from port. Depth greatly influenced density (Figure 4c–f), decreasing to up to approximately 18 m with a subsequent increase to approximately 30 m (but where few vessels occurred). Fitted significant models showed a complex response of the density of jigs with the distance to the PPA during Year 1 (Figure 4g), when only the current FPA1 was enforced with a partial protection status.

Habitat significantly influenced density in the Before (p<0.001), Year 2 (p<0.05) and Year 3 (p<0.05) periods, but was marginally non-significant in Year 1 (p = 0.06) and no relation was detected in the After period. Similarly to the other fishing gears, when aggregating all periods, the density of jigs was highly influenced by habitat (Kruskal-Wallis chi-square = 26.9211, p<0.001), with significantly (p<0.05) higher values in rocky reefs comparing to sand and mud.

### Hotspot Analysis and Individual Vessels Trends

**Figure 5:**
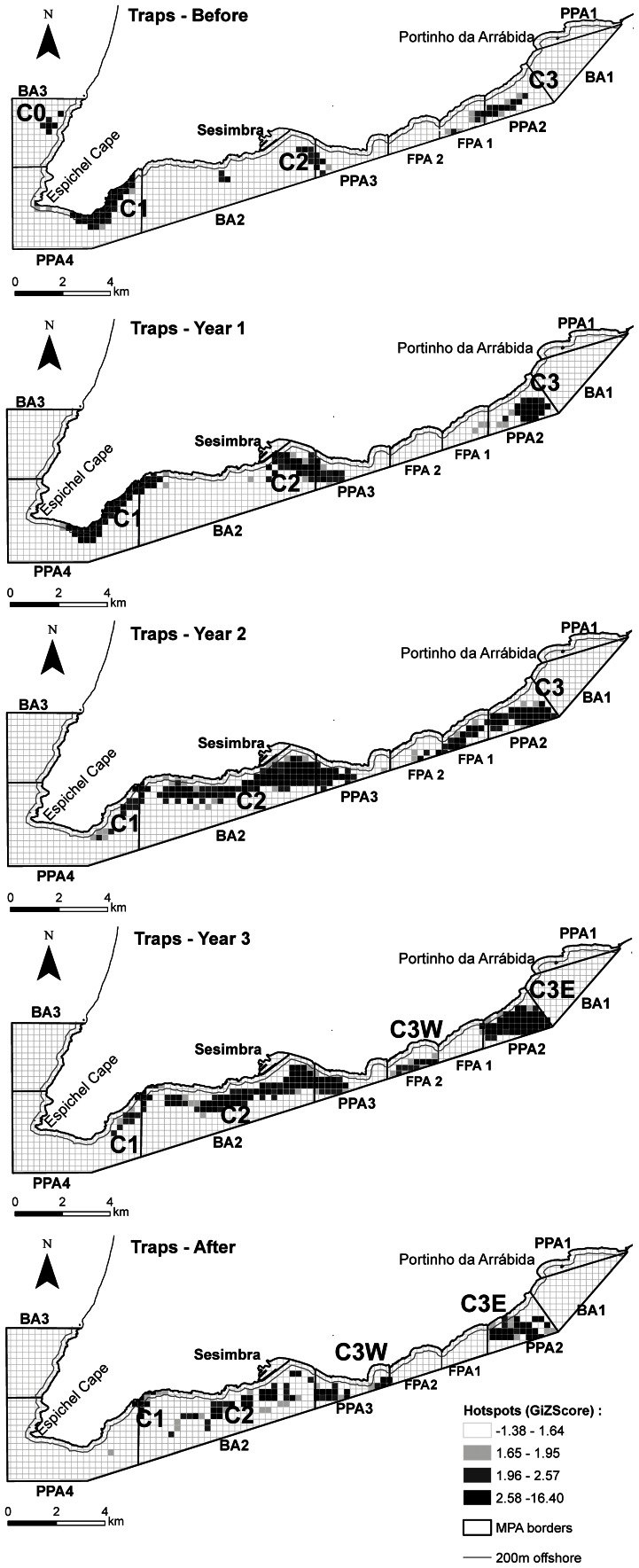
Maps obtained from the hotspot analyses of trap buoys for each period. The location of significant clusters (GIZScore >1.96) by period (a – Before, b – Year 1, c – Year 2, d – Year 3, e – After), and the different protection levels at the Arrábida Marine Park are shown: BA – buffer area; PPA – partial protection area; FPA – fully protected area (see Methods for a detailed description of the protection levels in the park and their implementation through time). The 200 m offshore line is also shown.

**Figure 6:**
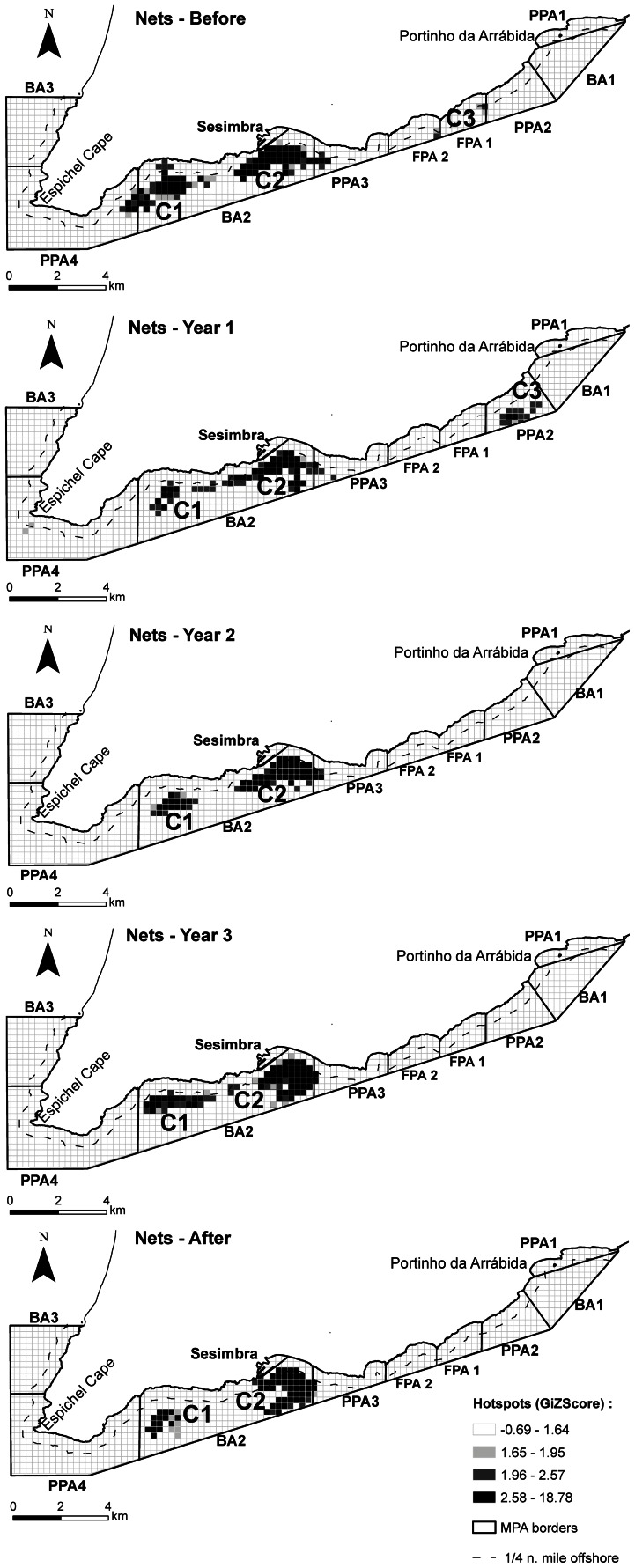
Maps obtained from the hotspot analyses of nets buoys for each period. The location of significant clusters (GIZScore >1.96) by period (a – Before, b – Year 1, c – Year 2, d – Year 3, e – After), and the different protection levels at the Arrábida Marine Park are shown: BA – buffer area; PPA – partial protection area; FPA – fully protected area (see Methods for a detailed description of the protection levels in the park and their implementation through time). The national legal limit for nets of the line of ¼ nautical miles offshore is also shown.

**Figure 7:**
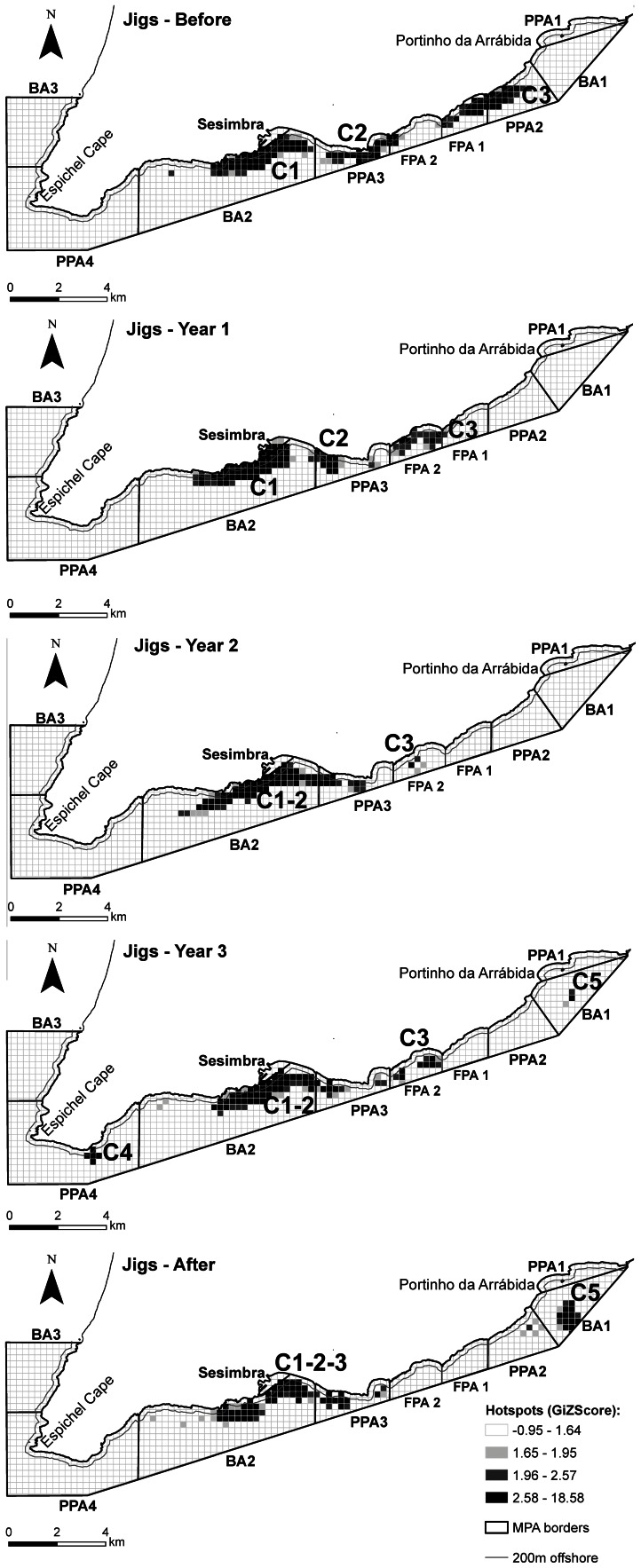
Maps obtained from the hotspot analyses of jig vessels for each period. The location of significant clusters (GIZScore >1.96) by period (a – Before, b – Year 1, c – Year 2, d – Year 3, e – After), and the different protection levels at the Arrábida Marine Park are shown: BA – buffer area; PPA – partial protection area; FPA – fully protected area (see Methods for a detailed description of the protection levels in the park and their implementation through time). The 200 m offshore line is also shown.

The hotspot analysis revealed the dynamics of significant fishing clusters throughout the different time periods and gear types analysed (Text S1). Traps followed closely the sequential enforcement of rules through the Implementation years, with some fishing effort displaced from no-fishing areas as shown in the rearrangement of clusters, some of which merged as a result of the MPA rezoning (Figure 5). On the other hand, the cluster closer to the no-take area was divided in two, with vessels surrounding its borders. The same rearrangement of clusters and changes in preferred areas (Text S1) as a consequence of the management plan implementation were also detected in nets (Figure 6) and jigs (Figure 7), although nets remained relatively stable through time in their main fishing grounds, which were already in fished areas. Jigs showed larger changes, with vessels generally moving towards home port but keeping close to the no-take zone. The contribution of individual vessels to each cluster in each time period was also analysed (Text S1) for traps (Figure S1), nets (Figure S2) and jigs (Figure S3).

## Discussion

Here we found that artisanal fisheries showed fisher- and fisheries-specific adaptations to multiple protection measures in a marine protected area (MPA). These findings suggest that artisanal fisheries from temperate systems have complex dynamics and that accounting for individual fishers’ behaviour and preferences in exploiting fishing grounds is crucial to implement more successful and effective multiple-use MPAs (i.e. areas with different zones with different types of rules applied to uses).

Different fisheries responded differently to the implementation of protection measures, with preferred habitats of target species driving fishers’ preferences in the selection of fishing grounds. Moreover, within each fishery individual fishers showed distinct strategies, with some operating in a broader area whereas others kept preferred territories, some of them being adjacent to a no-take area. Spatial allocation of fishing grounds was well defined and apparently agreed upon among the most common fishers, supporting the occurrence of traditional routines. One of the possible consequences of effort reallocation inside multiple-use MPAs is an increase of spatial competition for setting fishing gears in buffer areas [22]. When fishing effort is very high, the catchability of each gear may be reduced, affecting the expected benefits from protection. Interestingly, when fishers have licences for multiple gears, adapting to management rules may be easier. In fact, in our study traps and jigs faced a smaller reduction of fishing grounds than nets, although jigs may have lost important areas close to shore. Several fishers can opt to operate with various gears with a preference for traps instead of nets, as revealed by the increasing trend in the number of vessels fishing with traps. This suggests fishing with traps was the least affected fishery and that fishers are adapting to other productive alternatives in response to the zoning and rules of the marine park.

Some recent studies addressed the allocation of fisheries before and in response to spatial closures (temperate trawl fisheries: [14], [24]; tropical artisanal fisheries: [21], [22]), although we could not find other empirical cases in the literature where artisanal fishers’ distribution were analysed through direct observations before, during and after the implementation of a temperate MPA. Tracking the spatial position of vessels and fishing gears through time and analysing factors affecting the selection of a fishing ground may allow for a clearer understanding of the fishers’ choices and adaptations to different situations as well as of the dynamics of small scale artisanal fisheries, which comprise a large percentage of the fishing communities throughout the world.

Jigging for cuttlefish (*Sepia officinalis*) and squid (*Loligo vulgaris*) from small wooden vessels is a traditional artisanal fishing activity in the region. Jigging takes place mainly close to shore and near rocky reefs at depths up to 20 m. Thus, the jigging effort distribution in shallow areas can be attributable to species occurrence, gear characteristics and safety for these small vessels. Jigs were mainly influenced by depth and habitat through time. They were significantly more associated to rocky reefs than to other habitat types. Some previously preferred fishing grounds located inside the reserve may have become off-limits to these fishers since association with nearshore habitats lost significance with time and there was an important effect of the FPA location on vessels’ density before its implementation. Consequently, this fishery seems to have been impacted by the zoning as the fishers lost fishing grounds close to shore within the full and partially-protected areas. This may have negative consequences on the acceptance by fishers and on their attitudes towards the marine park [34].

A highly dynamic spatial distribution of jig vessels through time was detected with three main clusters identified. These were typically formed by a high number of vessels, sometimes with a large contribution of occasional fishers. In the After period, the three clusters that were previously scattered throughout the park merged into a single large cluster in front of the port where no restrictions apply to this fishery. The management plan implementation therefore caused some significant changes to the spatial distribution of this type of fishery, operated by small 3–4 m vessels, which take advantage of the very sheltered conditions of this coastline, mainly in front and to the east of their home port. They operate by drifting with the alongshore tides and target cephalopod species which occur in nearshore environments.

Benefits from protection may however have occurred since jig fishers remained in the area beyond 200 m in the PPAs, and near the western border of the FPA, which was the closest to their home port, even during the implementation phase of the fully protected area. This suggests that some fishers were able to profit by staying a little further away from shore, probably intercepting species over sandy bottoms adjacent to the shallow rocky reefs, rather than competing with other commercial and recreational fishers in the buffer zone.

Jig fishers’ adaptations suggest they tried to keep as close as possible to their former fishing ground, possibly also benefiting from protection, whereas at the same time their displacement was towards their home port, revealing other additional concerns probably related to security and operating costs. Lédée et al. [22] found that fishers preferentially redistributed to areas already known before protection, suggesting that previous experience and tradition may play an important role in the site-fidelity behaviour, influencing the choice of a fishing location. However, similar to the present study, the authors also report that most of the fishers’ displacement was towards their home port, mainly due to the lower costs, leading to an increase in the fishing pressure in areas that had already high density.

There were several factors explaining the spatial and temporal distribution of nets. Distance to port influenced effort density except in Year 3. The two main clusters occurred right in front of and to the west of the port and remained stable through time. A third cluster was detected in the initial periods in the east of the park encompassing part of the fully protected area but disappeared thereafter, with some fishing activities probably moving adjacently to the southern limit of the fully protected area outside the marine park limits. The proximity to both the partial and fully protected areas became important in the After period, with nets being located further away from these areas, which is consistent with the location of the main clusters.

The area in front and to the west of vessels’ home port is an important fishing ground where the main clusters consisting of several vessels were detected. Those clusters did not relocate after protection started as they were already in an allowed area. This is an extensive shallow sandy area used by commercial fishers targeting soles, cuttlefish and fish species such as sparids by trammel and gill nets [35]. When all periods were combined, buoy density was significantly associated with rock suggesting that fishers prefer shallow habitats, especially those with the potential to attract fish such as rocky outcrops and adjacent sand or shallow reefs.

The trap fishery showed preferred sites with clusters close to the home port and on each side of the park. Depth was the strongest influence in trap allocation with higher densities in shallow waters (18–20 m) and at around 70 m (but with fewer vessels), suggesting the possibility that these fishers were targeting different habitats. There were more traps distributed on sand than on other habitats, except for shallow rocky reefs. This is consistent with the behaviour of octopus (*Octopus vulgaris*, Cuvier, 1797), the target species of this gear, which is found in mixed sandy habitats, from the coastline to depths of around 200 m, usually spending the winter in deeper waters and migrating inshore by early spring to breed [36], [37].

The spatial dynamics of trap fishers showed a cluster close to their home port, which is advantageous for small vessels that cannot travel far for safety reasons, are limited by sea and weather conditions [20] and where operating costs are a significant burden. Another cluster was found near the most complex reefs of the park [38] which are also near the entrance of the Sado estuary, an important spawning and nursery area [39], with vessels extending their activity outside the park limits. Interestingly, the analysis of fishers’ choices through time showed that in this cluster (which in the Before period partially occupied the future fully protection area), considerable changes occurred in both the spatial distribution of traps and composition of vessels dominating this area. Although in Year 1 no cluster was found in the fully protection area (FPA), in Year 2 the eastern cluster extended to this area with fishers likely trying to gain access to this fishing ground before it became off-limits. This interpretation is reinforced by the fact that distance to the current FPA was not significant in the Before period, but became an important explanatory variable in the model during Year 3 and After periods (when the FPA was fully implemented), indicating that fishers were attracted to this area possibly due to the expectation of future benefits.

A few (3–5) vessels dominated several of these clusters and their behaviour changed through time. The western cluster became a hotspot dominated by a single vessel which was able to secure this fishing ground, whereas the central cluster was characterized by a larger number of vessels with a more erratic behaviour (i.e. vessels joined other clusters through time). This may be related to high competition in this fishing ground. On the other hand, with the retrieval of one dominant vessel that did not receive a license from the park, the eastern cluster was taken over by two new dominant vessels showing specific territories and dominance in these fishing grounds. These two vessels fished mainly on the borders of the FPA adopting a strategy of “fishing the line” [17]. Several reasons can explain this increase in effort at the edge of a no-take area: the reallocation and aggregation of effort because of the reduction of fishing grounds or due to perceived or expected benefits from protection [8], [40].

In spite of the loss of fishing ground as a consequence of MPA designation, the spatial competition between trap fisheries and, namely, nets decreased on important and traditional fishing grounds since nets became only allowed in the buffer areas. Moreover, before the management plan implementation, nearshore reefs were heavily exploited by spearfishing. The exclusion of this type of recreational fishery, which has a large impact on high trophic level species such as large sparids, seabass and octopus [41], [42], likely contributed to the increase of such target species’ biomass inside the marine park. In fact, the landings of octopus for vessels licensed to fish in the park have increased since protection started [43].

Other studies in Mediterranean MPAs found that the proximity to the reserve borders significantly affected the spatial distribution of fishing effort [15], [16], [20], [44]. The loss of fishing grounds and the attraction to the reserves’ boundaries when spillover effects are substantial, are important factors explaining the reallocation of fishing effort related to the implementation of MPAs. These effects are however influenced by the spatial distribution of habitats and target species inside and outside the reserve [45]. Thus, the proximity to no-take zones may not be involved in the choice of the fishing ground or may be due to the fishers’ preference for being closer to their former fishing location [22], [34].

Abesamis et al. [18] found that artisanal fishers tended to select traditional fishing grounds which were probably preferred due to their guarantee of higher stability in catches and a higher minimum average income. The experience and familiarity with fishing grounds, one component of traditional and ecological knowledge [46], [34], may also help to minimize gear loss and enhance catches.

### Conclusion

To understand the complexity of impacts (both positive and negative) on fisheries related to marine protected areas, one needs to closely follow the dynamics of fisheries operating nearby. This is particularly challenging for coastal multiple-use MPAs where artisanal fisheries occur. Here we show an effective method for the study of fishing effort allocation and dynamics for artisanal fisheries using different gears by following individual fishers’ choices before, during and after the implementation of protection. Our results have relevance to the vast majority of global MPA designs; that is, single, relatively small multiple-use areas utilized by local fishers using multiple gear types. Besides the importance of assessing fishing effort within and around MPAs, this study shows that gear type, habitat features and MPA design influence individual fishers’ behaviour and this must be taken into account when planning MPA design and evaluating the effects of marine conservation measures. This type of information is lacking in most studies evaluating the effects of marine protected areas although it is central for an unbiased assessment of biological, social and cultural responses to marine protection.

## Supporting Information

Figure S1 **Proportion contribution of vessels using traps (T) to each of the significant clusters obtained in the hotspot analysis of trap buoys by period: Before, Year 1, Year 2, Year 3, After.** See the location of each cluster in Fig. 5. The number of vessels observed in each cluster is also shown.
(TIF)

Figure S2 **Proportion contribution of vessels using nets (N) to each of the significant clusters obtained in the hotspot analysis of nets buoys by period: Before, Year 1, Year 2, Year 3, After.** See the location of each cluster in Fig. 6. The number of vessels observed in each cluster is also shown.
(TIF)

Figure S3 **Proportion contribution in of jig vessels (J) to each of the significant clusters obtained in the hotspot analysis of jig vessels by period (jigs were only correctly identified in Year 3 and After periods).** See the location of each cluster in Fig. 7. The number of vessels observed in each cluster is also shown.
(TIF)

Table S1 **Results of the smoothing terms from the generalized additive models (GAM) testing the density of trap buoys relative to the distance to several spatial features in the Before, implementation (Years 1, 2 and 3) and After periods.**
(DOC)

Table S2 **Results of the smoothing terms from the generalized additive models (GAM) testing the density of nets buoys relative to the distance to several spatial features in the Before, implementation (Years 1, 2 and 3) and After periods.**
(DOC)

Table S3 **Results of the smoothing terms from the generalized additive models (GAM) testing the density of jig vessels relative to the distance to several spatial features in the Before, implementation (Years 1, 2 and 3) and After periods.**
(DOC)

Text S1 **Hotspot analysis and individual vessels trends.** Analysis of significant clusters for each fishing gear type in the different protection areas throughout the Before, Implementation and After periods. Contribution of individual vessels for the observed trends in each cluster.
(DOC)
