# Supplementary figures and images for: Fishers’ Behaviour in Response to the Implementation of a Marine Protected Area
Source: PLoS One. 2013 Jun 3;8(6):e65057. doi: 10.1371/journal.pone.0065057 (PMC3670923; doi:10.1371/journal.pone.0065057)

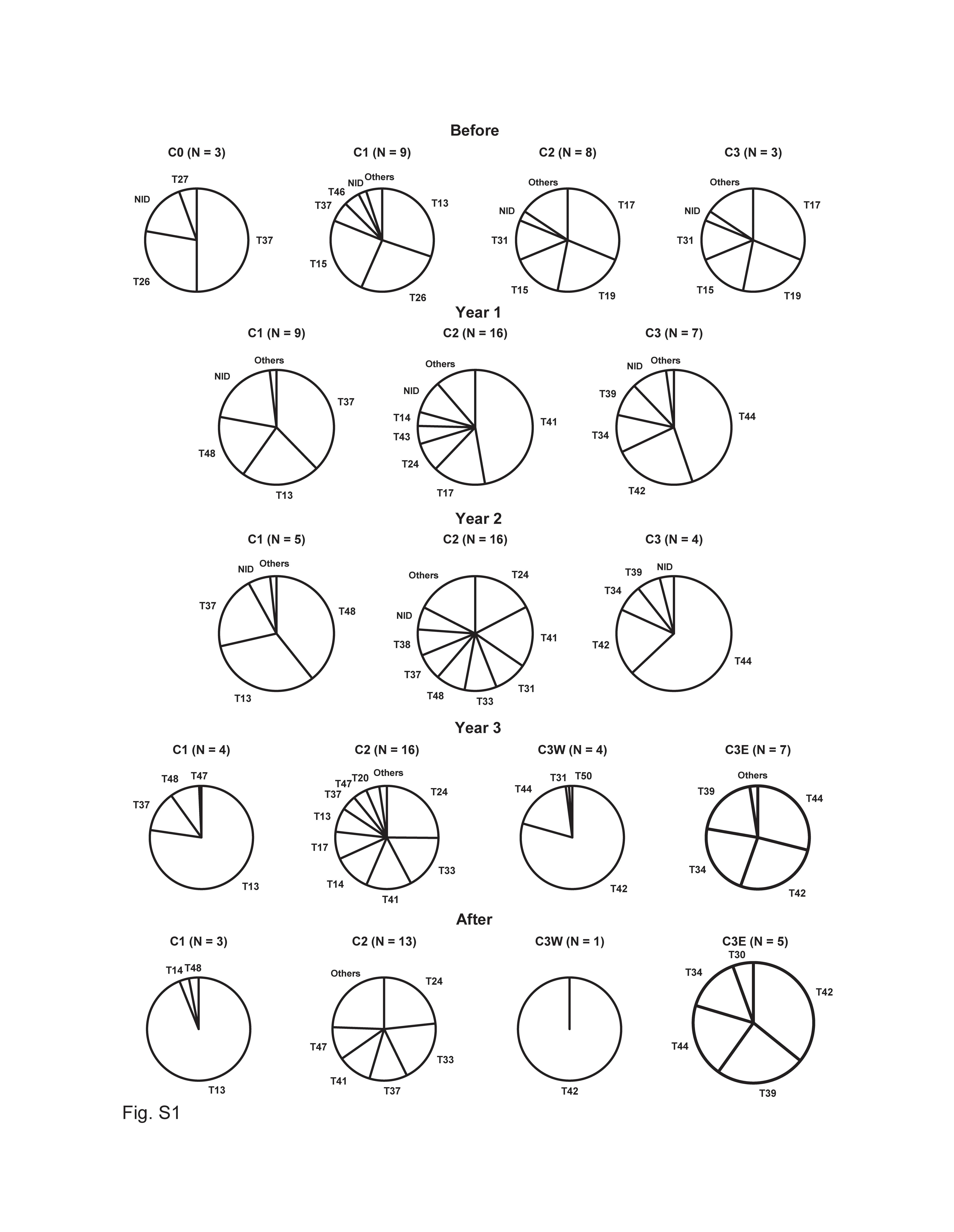

Supplement: Figure S1 — Proportion contribution of vessels using traps (T) to each of the significant clusters obtained in the hotspot analysis of trap buoys by period: Before, Year 1, Year 2, Year 3, After. See the location of each cluster in Fig. 5. The number of vessels observed in each cluster is also shown. (TIF) [file pone.0065057.s001.tif]

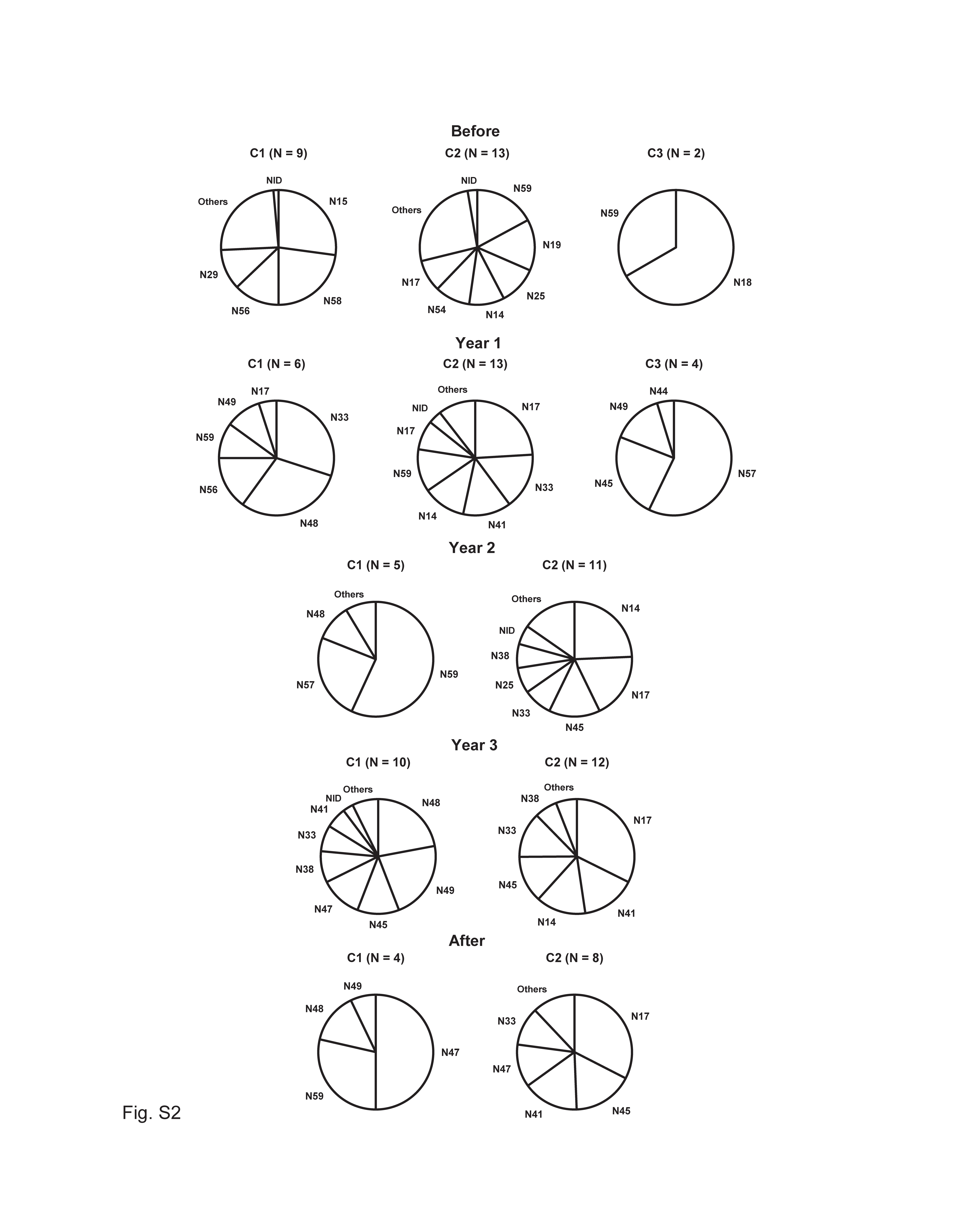

Supplement: Figure S2 — Proportion contribution of vessels using nets (N) to each of the significant clusters obtained in the hotspot analysis of nets buoys by period: Before, Year 1, Year 2, Year 3, After. See the location of each cluster in Fig. 6. The number of vessels observed in each cluster is also shown. (TIF) [file pone.0065057.s002.tif]

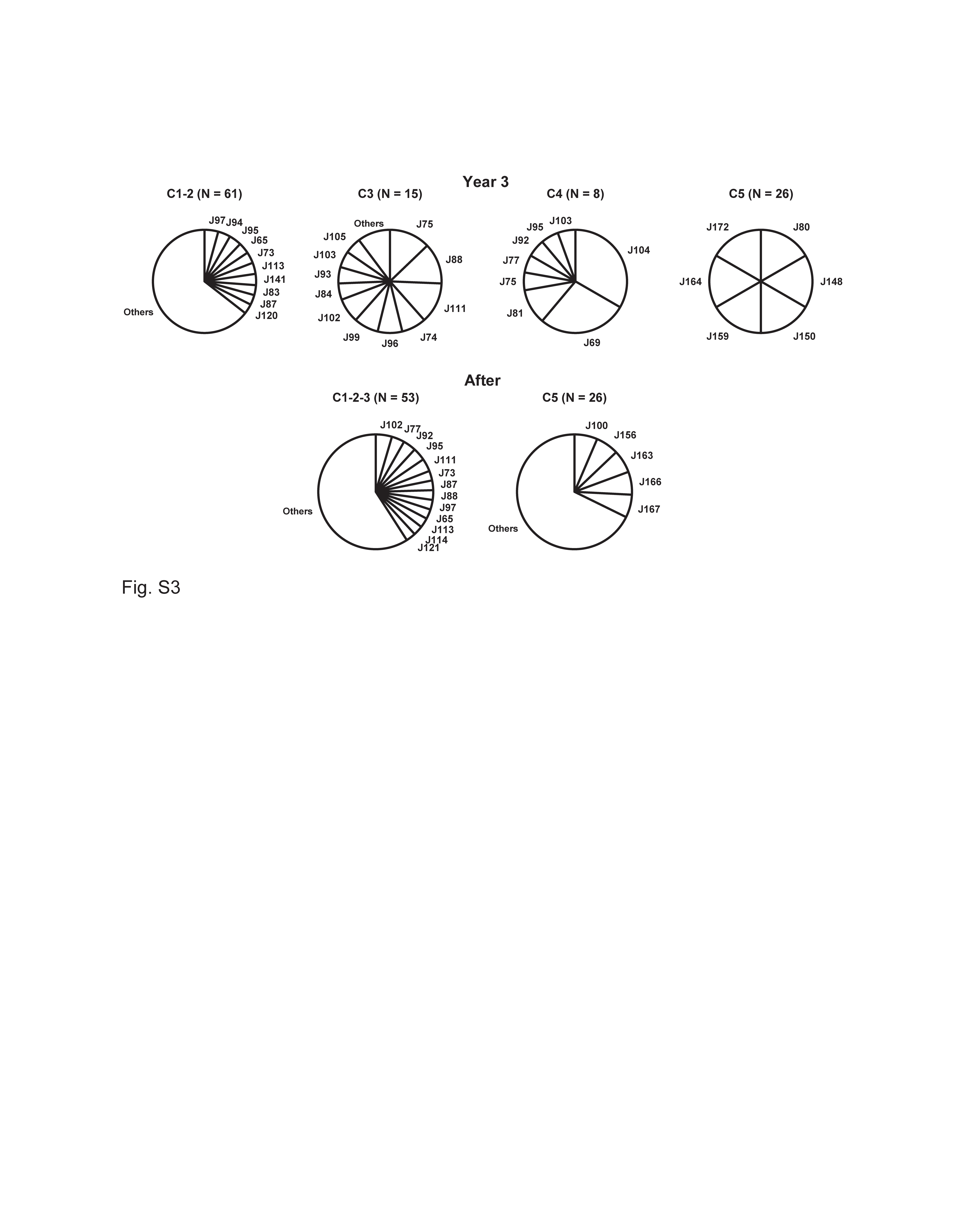

Supplement: Figure S3 — Proportion contribution in of jig vessels (J) to each of the significant clusters obtained in the hotspot analysis of jig vessels by period (jigs were only correctly identified in Year 3 and After periods). See the location of each cluster in Fig. 7. The number of vessels observed in each cluster is also shown. (TIF) [file pone.0065057.s003.tif]
