# Supplementary material for: Fishers’ Behaviour in Response to the Implementation of a Marine Protected Area
Source: PLoS One. 2013 Jun 3;8(6):e65057. doi: 10.1371/journal.pone.0065057 (PMC3670923; doi:10.1371/journal.pone.0065057)
Supplement: Table S3 — Results of the smoothing terms from the generalized additive models (GAM) testing the density of jig vessels relative to the distance to several spatial features in the Before, implementation (Years 1, 2 and 3) and After periods. (DOC) [file pone.0065057.s006.doc]

Table S3 – Results of the smoothing terms from the generalized additive models (GAM) testing the density of jig vessels relative to the distance to several spatial features in the Before, implementation (Years 1, 2 and 3) and After periods.

| **Period** | **Explanatory variables** | **edf** | **F** | **p-value** | **Deviance explained (%)** |
| --- | --- | --- | --- | --- | --- |
| Before | s(DistSes) | 7.12 | 2.663 | **p < 0.05** | 56.60 |
|  | s(Depth) | 5.091 | 6.832 | **p < 0.001** |  |
| Year 1 | s(DistSes) | 1 | 1.355 | n.s. | 84.40 |
|  | s(Depth) | 4.545 | 2.238 | p = 0.085 |  |
|  | s(Dist_PPA) | 8.234 | 2.637 | **p < 0.05** |  |
| Year 2 | s(DistSes) | 1 | 38.814 | **p < 0.001** | 61.80 |
|  | s(Depth) | 4.53 | 6.829 | **p < 0.001** |  |
|  | s(Dist_PPA) | 1.343 | 2.839 | p = 0.075 |  |
| Year 3 | s(DistSes) | 5.523 | 1.321 | n.s. | 65.90 |
|  | s(Depth) | 6.478 | 6.058 | **p < 0.001** |  |
|  | s(Dist_PPA) | 1.885 | 1.153 | n.s. |  |
|  | s(Dist_FPA) | 2.502 | 0.955 | n.s. |  |
| After | s(DistSes) | 1.281 | 1.013 | n.s. | 65.80 |
|  | s(Depth) | 6.306 | 5.967 | **p < 0.001** |  |
|  | s(Dist_PPA) | 2.585 | 0.856 | n.s. |  |
|  | s(Dist_FPA) | 6.01 | 1.651 | n.s. |  |

Explanatory variables selected are: distance to Sesimbra port (DistSes), depth, distance to PPA (DistPPA), distance to FPA (DistFPA). Estimated degrees of freedom (edf), F-statistics and corresponding p-values are indicated. Significant values are in bold; n.s. = non-significant; marginally non-significant values are also shown.
