# Supplementary material for: Fishers’ Behaviour in Response to the Implementation of a Marine Protected Area
Source: PLoS One. 2013 Jun 3;8(6):e65057. doi: 10.1371/journal.pone.0065057 (PMC3670923; doi:10.1371/journal.pone.0065057)
Supplement: Text S1 — Hotspot analysis and individual vessels trends. Analysis of significant clusters for each fishing gear type in the different protection areas throughout the Before, Implementation and After periods. Contribution of individual vessels for the observed trends in each cluster. (DOC) [file pone.0065057.s007.doc]

**Supplementary information**

**Text S1** - Hotspot analysis and individual vessels trends

*Traps*

The hotspot analysis of trap distribution revealed three significant clusters common to all periods and a fourth one north of the Espichel cape (Figure 5). The latter (the north cluster – C0) is exclusive to the Before period since this area was only assessed then. The other three are the western cluster (C1), located south-east of the Espichel cape, the central cluster (C2), in front of Sesimbra port, and the eastern cluster (C3) occupying mainly the current PPA2. In Year 2, when all PPAs started and traps were allowed only beyond 200 m from shore (except in the BAs), the western cluster (C1) shifted and merged with the central cluster (C2) which then occupied a large part of BA2. The eastern cluster (C3) occupied the PPA2 in the Before and Year 1 periods and a portion of the FPA1 (not statistically significant in Year 1). In Year 2 this cluster increased in size and extended well into the FPA1 (still a PPA at that time). In Year 3, the FPA1 was enforced with full protection status and this cluster divided in two parts which respectively occupied the FPA2 and PPA2. In the After period, when both FPA1 and FPA2 were enforced, the part of the cluster which occupied the FPA2 in the previous period moved to the eastern border of PPA3 (adjacent to the fully protected area).

The contribution of individual vessels to each cluster in each time period was analysed (Figure S1). Some important vessels from the Before period shift location or disappeared after that. The number of vessels in the cluster C1 decreased with time, with one vessel (T13) increasing their contribution throughout periods and dominating that hotspot in the After period (94%). The central cluster (C2) contained a large number of vessels changing their relative importance through time with only two vessels (T24 and T41) occurring in all periods (except in the Before period). The eastern cluster (C3) was initially dominated by vessel T18 (85%) which also disappeared from the park after this period (this vessel was not granted a license to fish in the park). Vessels T42 and T44 started to dominate this cluster after Year 1 and were joined by two additional vessels (T34 and T39). Interestingly, in Year 3 this cluster was split in half with the enforcement of the eastern half of the FPA, with vessels T42 (79%) and T44 (19%) dominating the FPA2 area (C3W) (Figure S1). In the After period, T42 was the only vessel responsible for the significance of this cluster, which was located adjacent to the western limit of the FPA. In the eastern half of the cluster (C3E), all four vessels were equally contributing to this aggregation both in Year 3 and the After period.

*Nets*

Three significant clusters were obtained from the hotspot analysis in the Before and Year 1 periods, but the eastern cluster (C3) located in FPA1 (Before) and in PPA2 (Year 1) disappeared (Figure 6). The two large clusters located in the central (C2) and western (C1) part of the park remained relatively stable through time. Significant clusters were generally beyond the legal limit of ¼ nautical miles for nets.

The western cluster (C1) had important contributions from vessels that were only present in the park before the management plan implementation (N15, N29, N56, N58). After that, vessel N48 was present in all periods and several other vessels contributed significantly to this cluster (Figure S2). The main vessels contributing to the central cluster (C2) remained stable over time (N14, N17, N33). Several vessels changed between these clusters both within the same period and between periods (Figure S2).

*Jigs*

The hotspot analysis showed varying numbers of significant clusters in the different periods (Figure 7). Three clusters remained relatively stable through time. A western cluster (C1) located very close to shore immediately to the west and in front of Sesimbra port, merged with a central cluster (C2) located to the east of Sesimbra in Year 2, forming thereafter a single cluster. The eastern cluster (C3) was located in FPA1 and PPA2 in the Before period but it moved closer to Sesimbra after that, first to FPA2 and PPA3 in Years 1, 2 and 3, merging with the cluster C1-2 in the After period although it remained adjacent to the FPA border. In Year 3 two new significant clusters formed in the park: one near Espichel cape (C4) and one close to the Portinho da Arrábida bay (C5). However, the former did not remain significant in the After period whereas the latter increased in density.

The analysis of individual vessel contributions to each cluster was only conducted for Year 3 and After periods (when information of individual vessels was collected for this gear type) (Figure S3). In Year 3, the merged western (C1) and central (C2) clusters contained a high number of vessels (n = 61) detected. The eastern cluster (C3) also contained several vessels (n = 15) but with a lower percent contribution of less frequent (occasional) fishers. The Espichel cluster (C4) contained eight contributing vessels with two main fishers (J69, J104) influencing this distribution, whereas the Portinho cluster (C5) had six vessels that were only seen once and thus their contribution was even. Although the Espichel cluster was only detected in Year 3, this was however a very important area for longlines which are operated by similar vessels and so fishers found jigging could have previously been fishing with longlines (which are only allowed in the buffer area). In the After period, the three merged clusters (C1-2-3) contained fewer vessels (n = 53) than in the previous period but again with a high proportion of occasional fishers. The Portinho cluster had a larger number of vessels (n = 26) although these were not detected in the previous period (Figure S3).
